# The study of automatic machine learning base on radiomics of non-focus area in the first chest CT of different clinical types of COVID-19 pneumonia

**DOI:** 10.1038/s41598-020-76141-y

**Published:** 2020-11-03

**Authors:** Hui-Bin Tan, Fei Xiong, Yuan-Liang Jiang, Wen-Cai Huang, Ye Wang, Han-Han Li, Tao You, Ting-Ting Fu, Ran Lu, Bi-Wen Peng

**Affiliations:** 1 Department of Radiology, PLA Central Theater General Hospital of Chinese, Wuhan, China; 2 School of Basic Medical Sciences, Wuhan University, Wuhan, China

**Keywords:** Outcomes research, Infectious diseases, Respiratory tract diseases

## Abstract

To explore the possibility of predicting the clinical types of Corona-Virus-Disease-2019 (COVID-19) pneumonia by analyzing the non-focus area of the lung in the first chest CT image of patients with COVID-19 by using automatic machine learning (Auto-ML). 136 moderate and 83 severe patients were selected from the patients with COVID-19 pneumonia. The clinical and laboratory data were collected for statistical analysis. The texture features of the Non-focus area of the first chest CT of patients with COVID-19 pneumonia were extracted, and then the classification model of the first chest CT of COVID-19 pneumonia was constructed by using these texture features based on the Auto-ML method of radiomics, The area under curve(AUC), true positive rate(TPR), true negative rate (TNR), positive predictive value(PPV) and negative predictive value (NPV) of the operating characteristic curve (ROC) were used to evaluate the accuracy of the first chest CT image classification model in patients with COVID-19 pneumonia. The TPR, TNR, PPV, NPV and AUC of the training cohort and test cohort of the moderate group and the control group, the severe group and the control group, the moderate group and the severe group were all greater than 95% and 0.95 respectively. The non-focus area of the first CT image of COVID-19 pneumonia has obvious difference in different clinical types. The AUTO-ML classification model of Radiomics based on this difference can be used to predict the clinical types of COVID-19 pneumonia.

## Introduction

Since January 2020, pneumonia caused by novel coronavirus broke out in Wuhan, China, it was named COVID-19 by world health organization (WHO). COVID-19 is a kind of ribonucleic acid virus mainly transmitted through respiratory tract. The main harm of COVID-19 pneumonia is to cause adult acute respiratory distress syndrome (ARDS). COVID-19 virus can be detected in respiratory tract like severe acute respiratory syndrome (SARS) virus^1,2^. By the end of February, it has been extended to over 100 countries worldwide. It is estimated that more than 50,000 patients have been diagnosed with over 2500 deaths. Studies showed that early effective treatment can significantly block the course of disease and reduce the conversion rate of critical illness^3^. Therefore, it is necessary to use effective methods to detect lung lesions in patients with COVID-19 pneumonia^4–6^.

The common clinical symptoms of COVID-19 pneumonia include fever, cough, sore throat, occasional chest tightness, expectoration, muscle soreness, etc., but these symptoms are not the same in the early stage of COVID-19 pneumonia, and these symptoms are not unique symptoms of covid-19 pneumonia. When the epidemiological history is not clear or the patient intentionally conceals the medical history, clinicians often treat the patients according to the suspected diagnosis, rather than the targeted treatment with clear diagnosis. Chest CT is an important method for the diagnosis of COVID-19 pneumonia, which is widely used in the diagnosis of COVID-19 pneumonia, to guide the adjustment of clinical treatment plan and verify the treatment effect^7,8^.

In the chest CT images, the typical manifestations of the focus of COVID-19 pneumonia are parapleural ground glass (GGO), interlobular septal thickening, central consolidation of the focus and banded atelectasis^8,9^. However, in the first CT examination of patients with COVID-19 pneumonia, the characteristics of the focus are often not typical, some of which cannot clearly be used to diagnose and classify the pneumonia of COVID-19, thus limited the value for clinical design of treatment plan.

The inflammatory reaction of interstitial and alveolar edema in Non-focus lung tissue during the early lung injury of COVID-19 pneumonia, which is difficult to be distinguished by eyes on CT images^4,9,10^. As an extension of computer-aided diagnosis, Lambin proposed the Radiomics method in 2012^11^. It will extract and analyze image texture features and combine them with other available patient data to enhance the ability of decision model. The method of Radiomics analysis can make the inflammatory reaction of alveolar interstitium and alveolar edema in the Non-focus area which is difficult to be distinguished by eyes in the early chest CT image of COVID-19 pneumonia become the image information that can be excavate and utilized.

Therefore, our aim is to establish and validate a prediction model of Non- focus area in the early stage of COVID-19 pneumonia by excavate the texture features of the first chest CT image with the method of Auto-ML, and to evaluate the value of the model in the degree of Non-focus area damage and clinical classification in the early stage of COVID-19 pneumonia.

## Methods

The study is based on the principles of the Helsinki declaration. The Ethics Committee of the PLA Central Theater General Hospital approved this study because it is a retrospective study, giving up the need for written informed consent (Decision/Protocol number: [2020]030-1).

### Patients selection

**Figure 1:**
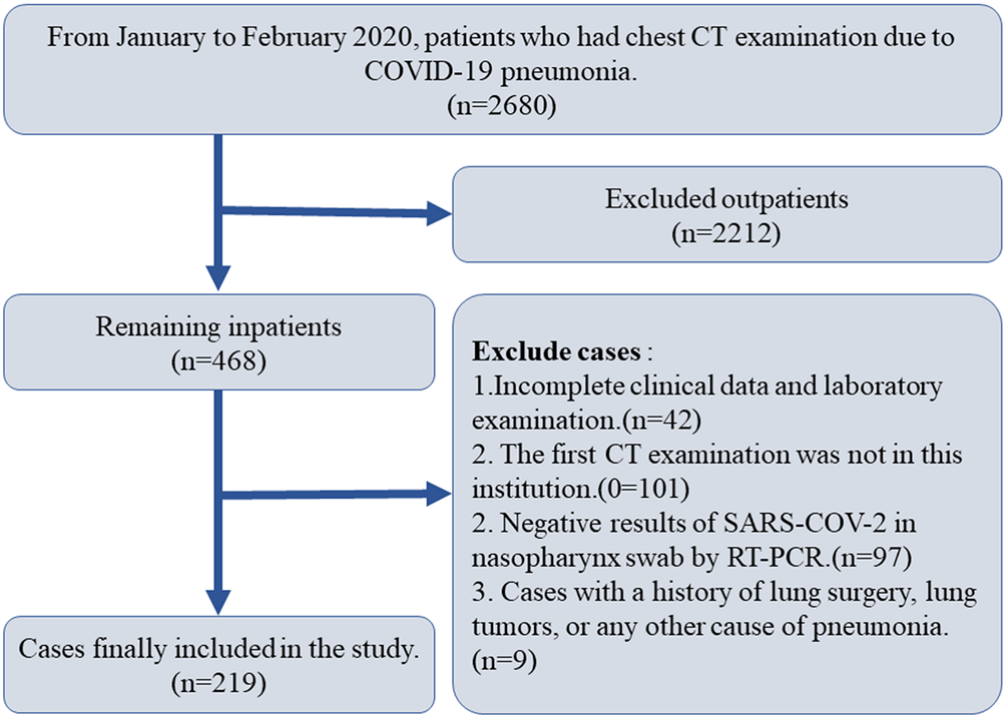
Flow chart of selecting case samples. Case elimination flowchart. 219 patients were selected according to the inclusion criteria.

**Figure 2:**
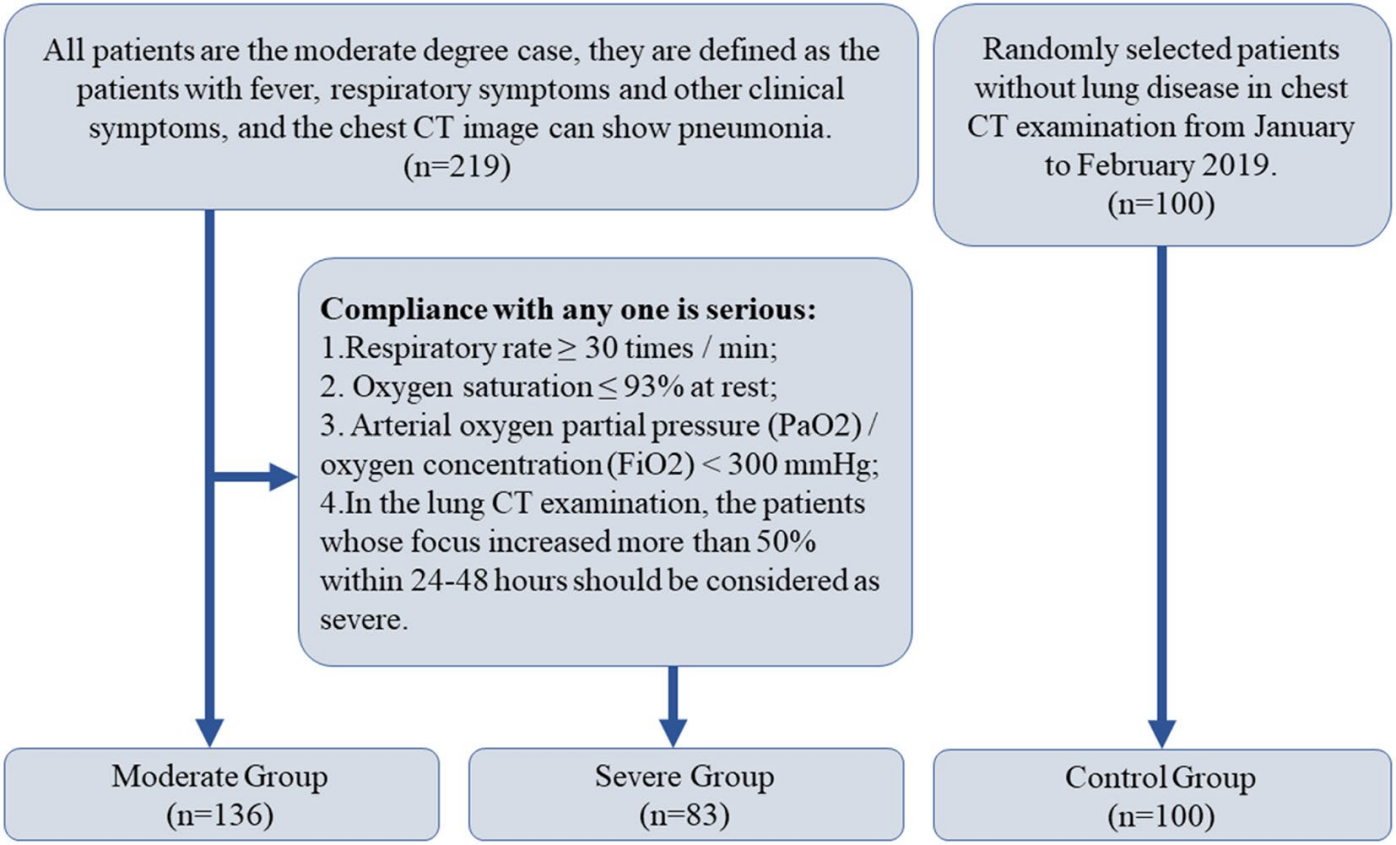
Flow chart of grouping case samples. Case Group Flowchart. 219 patients were divided into groups according to the COVID-19 diagnostic and therapeutic regimen (trial 7th edition) in China.

Collected 2680 patients with COVID-19 pneumonia diagnosed according to the COVID-19 diagnostic and therapeutic regimen (trial 7th edition) in China (www.nhc.Gov.cn/yzygj/s7652m/202003/a31191442e29474b98bfed5579d5af95.shtml), From January 2020 to February 2020. They were included in the study according to the following conditions: 1. Hospital patients. 2. The clinical information and laboratory examination were complete, and at least two lung CT examinations (including the first CT examination) were performed within one week after hospitalization. 3. Positive results of severe acute respiratory syndrome coronavirus 2 (SARS-COV-2) in nasopharynx swab by RT-PCR. 4. Cases with a history of lung surgery, lung tumors, or any other cause of pneumonia were excluded. Finally, 219 patients were included in the study (Fig. 1). In order to prevent asymptomatic cases infected with COVID-19 virus from being added to the control group, we randomly selected 100 cases from the physical examination population who had chest CT examination and no lung lesions between January and February 2019 as the control group (Fig. 2).

Clinical characteristics, including age, gender, temperature, cough, sputum, nausea and vomiting and other clinical symptoms; white blood cells (WBC), lymphocytes, alanine aminotransferase (ALT), aspartate aminotransferase^12^, C-reactive protein (CRP), fibrinogen, urea (URE), creatinine (CRE) were obtained from the medical records, see Table 1. The clinical symptoms were the symptoms at the time of admission, and blood samples were taken for examination within 3 days after admission.

**Table 1:** Patients with clinical data and Statistics results.

Characteristics	All cases (n = 319)			
	Severe (n = 83)	Moderate (n = 136)	Control (n = 100)	P-value^#^
**General data**				
Age	49.46 ± 11.95	51.86 ± 13.63	50.96 ± 11.20	0.382
Gender				
Male (female)	48(35)	79(57)	57(43)	0.078
Smoke				
Yes (No)	21(62)	30(106)	31(69)	2.422
**Symptom**				
Temperature (℃)				
> 37.3 (< 37.3)	26(57)	38(98)	(100)	0.593
Cough				
Yes (No)	63(20)	88(48)	4(96)	0.065
Sore throat				
Yes (No)	59(24)	94(42)	3(97)	0.674
Sputum				
Yes (No)	45(38)	79(57)	15(85)	0.575
Dyspnea				
Yes (No)	14(69)	25(111)	2(98)	0.000
Muscle soreness				
Yes (No)	54(29)	71(65)	0(100)	0.026
**Laboratory data**				
WBC (10^9^/L)				
4–10 (< 4 or > 10)	64(19)	95(41)	0(100)	0.199
Lymphocyte (10^9^/L)				
1.1–3.2(< 1.1)	28(55)	39(97)	100(0)	0.411
ALT (U/L)				
≤ 40(> 40)	78(5)	119(17)	100(0)	0.095
AST (U/L)				
≤ 40(> 40)	78(5)	117(19)	100(0)	0.070
CRP (mg/L)				
≤ 10(> 10)	23(50)	48(88)	100(0)	0.563
FIB (g/L)				
1.5–3.5 (> 3.5)	39 (44)	82(54)	100(0)	0.054
Ure (mmol/L)				
≤ 7.1(> 7.1)	67(16)	102(34)	100(0)	0.342
Cre (umol/L)				
≤ 106(> 106)	62(21)	101(35)	95(5)	0.968

^#^In symptom and laboratory data, the statistical values of the study group and the control group are significantly different, and the Chi square test only carries out the cross test within the study group.

**Figure 3:**
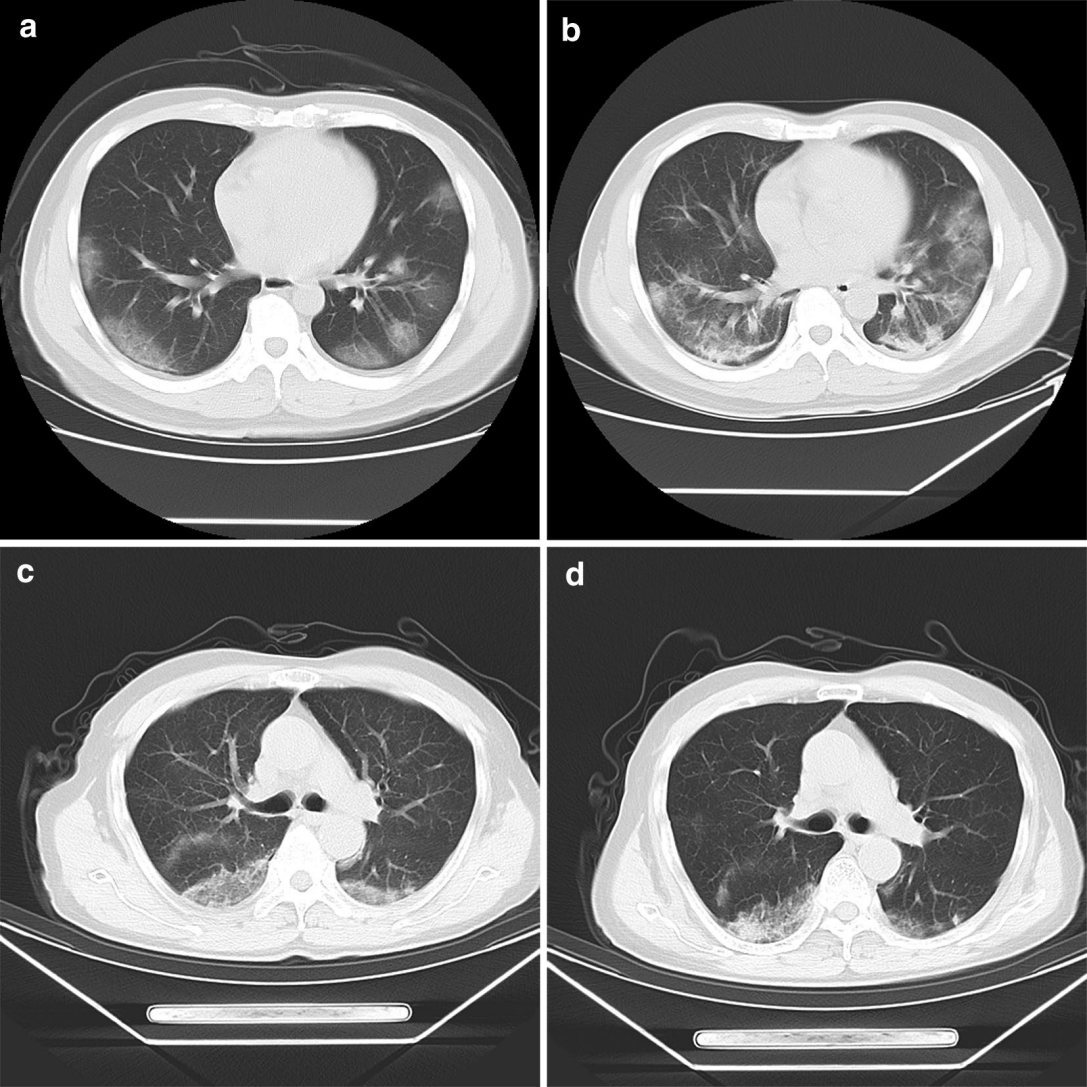
(**a**–**d**) Axial CT image, showing the changes of the first chest CT and the second chest CT image after 7 days of treatment in patients with “severe” and “moderate”, respectively. A 43-year-old male patient classified as “Severe”, showed the typical lesion level image of the first CT examination, and the subpleural areas of the two lungs are scattered with mass like ground glass shadow (**a**), showed the CT image of reexamination after 7 days of hospitalization treatment. The original lesion range is expanded, and the strip like consolidation area appears in the lesion (**b**); a 65-year-old man classified as “Moderate” was admitted to the hospital due to fatigue, headache and muscle ache. showed the typical focus level image of the first CT examination, and the focus similar to patient 1 can be seen, but the central area of the focus is partially solid (**c**), showed the CT image of reexamination 7 days after hospitalization, the original focus range of the two lungs is narrowed, the exudation part of the focus is absorbed, and the edge of the focus is clear (**d**).

According to the scheme of “COVID-19 diagnostic and therapeutic regimen (trial 7th edition) in China”, the moderate degree cases are defined as the patients with fever, respiratory symptoms and other clinical symptoms, and the chest image can show pneumonia. The severe cases were defined as adults who met any of the following criteria: respiratory rate ≥ 30 times/min; oxygen saturation ≤ 93% at rest; arterial oxygen partial pressure (PaO_2_)/oxygen concentration (FiO2) < 300 mmHg. In the lung CT examination, the patients whose focus increased more than 50% within 24–48 h should be considered as severe. All the 219 patients were of moderate degree at the time of admission, 83 of them developed to serious degree in 7–13 days after admission, and the other 136 cases were stable in the moderate degree (Figs. 2 and 3).

### CT image acquisition

Chest CT images were obtained with GE Lightspeed/16 slice CT scanner (GE Healthcare, Beijing). Scanning range: upper edge of cervical vertebra 7 to lumbar vertebrae 2. Scanning parameters: rotating speed of spherical tube 0.625 s/rot, pitch 1, field of view (FOV) 250, tube voltage 100-120kv, adaptive tube current technology (110mas-140mas). reconstruction parameters: matrix 512 * 512, 1.25 mm slice thickness and 1.25 mm interval, window level -550HU, window width 1500HU and average density projection mode.

### Image segmentation

**Figure 4:**
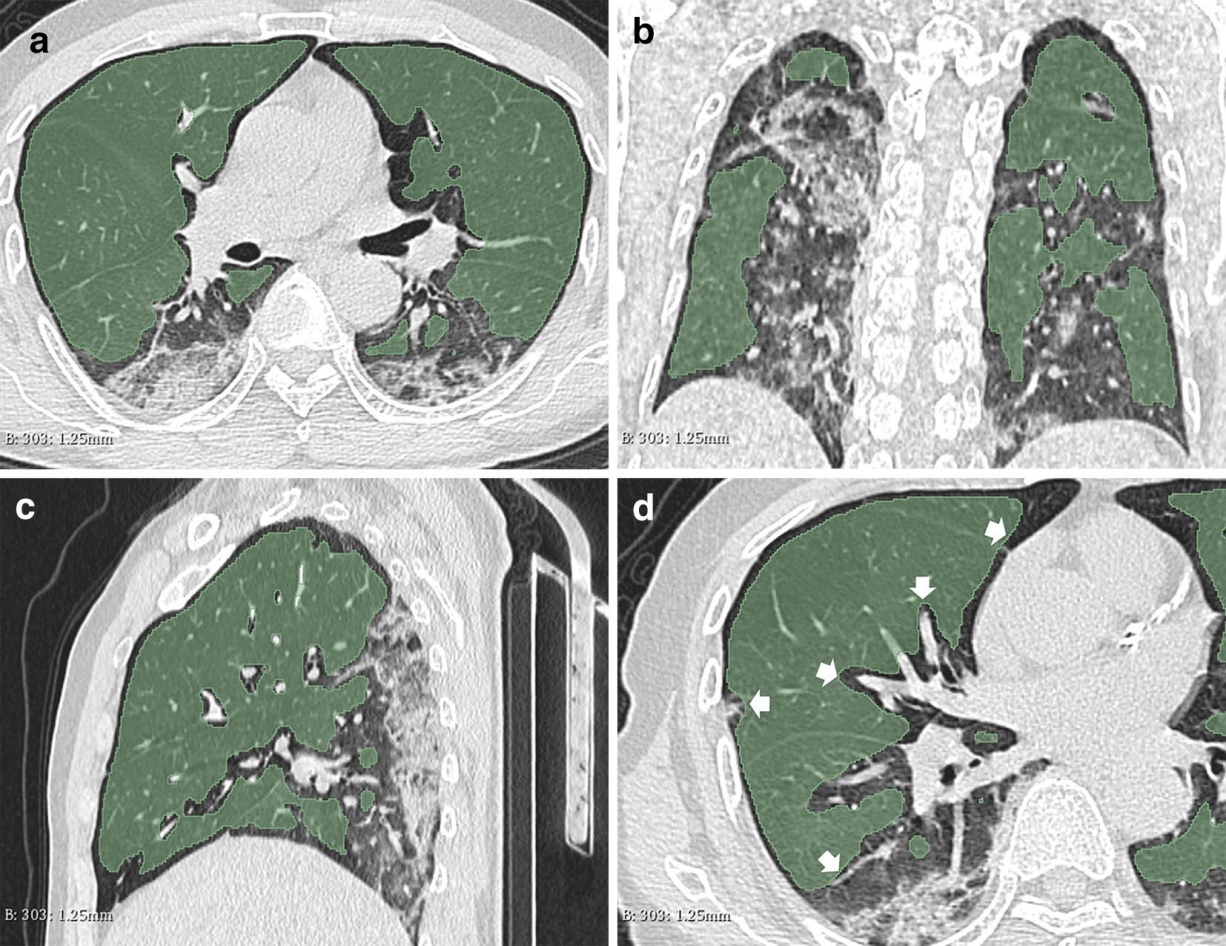
(**a**–**d**) Axial (**a**), coronal (**b**) and sagittal (**c**) of CT images, showing the VOI range of segmented images. The region growing method was used to segment VOI on 3D-slicer software. By adjusting the threshold range of CT value to exclude the pulmonary vessels and bronchi above the second grade in the lung, Gauss smoothing was used to reduce the edge of VOI by 3 mm, avoid overlapping with the focus and disturb the analysis results. Typical image layers of axial, coronal and sagittal plane of the same patient's CT image (**a**–**c**); “White arrow” marks the boundary of artificial shrinkage (**d**).

Study on the images of the first CT examination of the patients. All CT images were segmented by a free and open source 3D-Slicer (4.10.2 version) software (www.slicer.org) for semi-automatic image segmentation^13^. Firstly, take regional growth to draw the volume of interest (VOI) of the non-focus part of the lung, then two radiologists with more than 10 years of experience manually modified and shrunk the VOI edge to 3 mm from the focus edge. Data Supplement presents the VOI drawing methods and modification criteria (Fig. 4).

### Radiomics features extraction

In this study, we used Python language (version 3.7.4) program to call the Pyrometric package (version 2.2.0)^14,15^. In the process of program running, seven filters are used to process the original VOI. Seven categories (each category including: 18 features of first-order statistics (FOS), 24 features of gray level co-occurrence matrix (GLCM), 16 features of gray level run matrix (GLRLM), 16 features of gray level size region matrix (GLSZM) and 5 of neighbouring gray tone difference matrix (NGTDM) are extracted from each filtered image There are 1674 texture features. Together with 14 shape features of the original image, 1688 features were extracted for this study. For more information on the methods and parameters of feature extraction in radiomics^16^, see Table 2.

**Table 2:** The number of Radiomics features of CT images extracted with Pyradiomics2.2.0.

Filters	Category							Total
	FOS	GLCM	GLRLM	GLSZM	NGTDM	GLDM	Shape#	
Original image	18	24	16	16	5	14	14	107
Wavelet*	144	192	128	128	40	112	0	744
Square	18	24	16	16	5	14	0	93
Square Root	18	24	16	16	5	14	0	93
Logarithm	18	24	16	16	5	14	0	93
Exponential	18	24	16	16	5	14	0	93
Gradient	18	24	16	16	5	14	0	93
LocalBinaryPattern2D	18	24	16	16	5	14	0	93
LocalBinaryPattern3D*	54	72	48	48	15	72	0	279
Total	324	432	288	288	90	252	14	1688

Note: #Category” Shape” features were only extracted from the original image, were not included in the group analysis because of the obvious difference between the study group and the control group;

*Filter “Wavelet” uses 8 levels of LLL, LLH, LHL, LHH, HLL, HHL, HLH, HHH to process images;

*Filter “LocalBinaryPattern3D” uses 3 levels of m1, m2, and k to process images.

### Auto-ML

**Figure 5:**
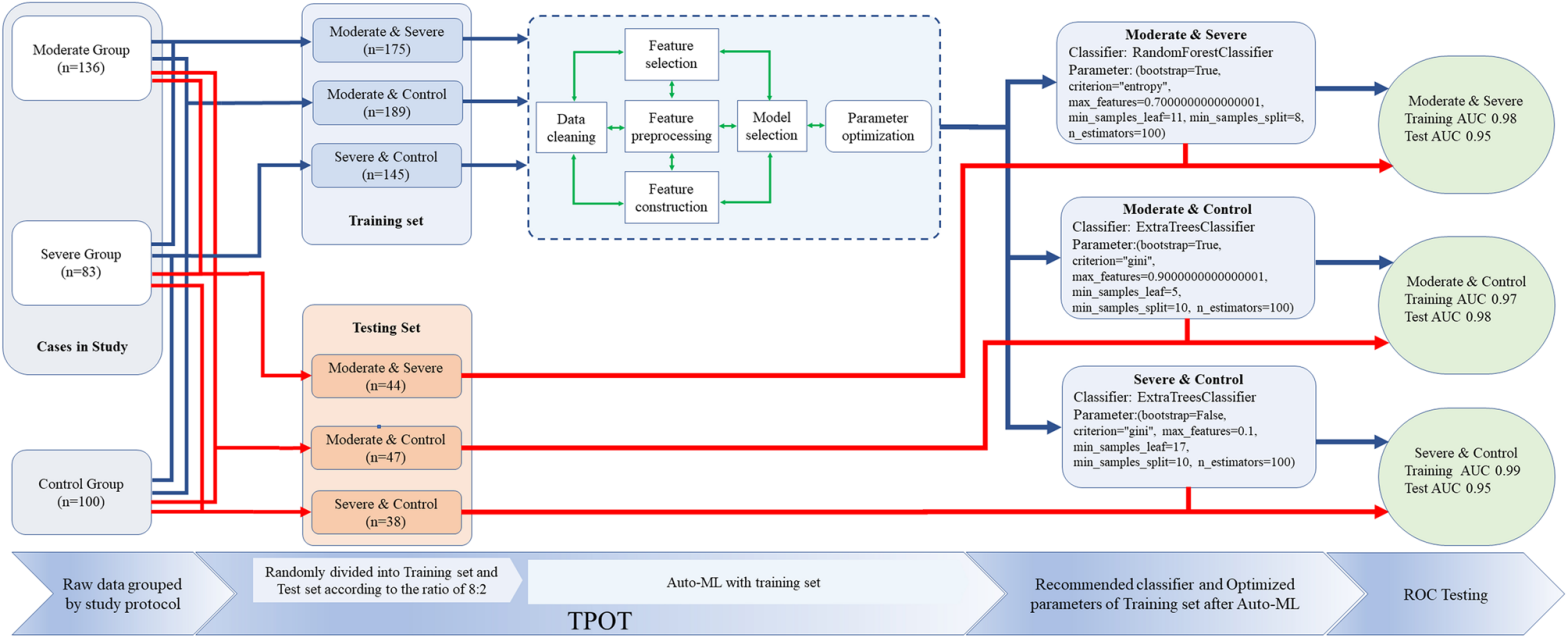
Workflow of TPOT pipeline. At the beginning of the pipeline, each group of original data was randomly divided into training set and test set according to the proportion of 8:2. In the pipeline, The training set was repeated for data cleaning, feature selection, feature construction, feature processing, model selection and parameter optimization, until a pipeline (classifier) optimized for the parameters of a specific model was selected. In the last stage of the pipeline, the data of the training set and the test set were mixed, then the data was put into the optimal pipeline to verify whether the pipeline and parameters were optimal. The specific operators selected in the best pipeline include the built-in TPOT operator (OneHotenCoder, FeatureSetSelector) and the functions in the scikitlearn library (ExtraTreesClassifier and RandomForstClassifier).

In the texture feature data, since the shape related parameters of the control group and the study group are significantly different, they are removed from the data matrix during the analysis. Tree-based pipeline optimization tool (TPOT) (epistasislab. github. io/tpot) is a python Auto-ML tool based on genetic algorithm to optimize Auto-ML pipeline^17–19^. In the process of Auto-ML, each group's original data is imported into TPOT, and TPOT randomly divides the original data into training set and test set according to the proportion of 8:2. In the Auto-ML process of training set, TPOT repeatedly carries out data cleaning, feature selection, feature preprocessing, feature construction, model selection and parameter optimization through intelligent exploration of thousands of possible pipeline, automatically realizes feature analysis of shadow parts, and carries out in training set verification. After the exploration and verification, the available Python code containing classifier information and corresponding parameter settings is generated (Fig. 5).

### Classification model testing

According to the results of TOPT analysis, classifier was selected and classifier parameters were set (generations = 5, population size = 20, verbosity = 2). Three models of Moderate and Severe group, Moderate and control group, Severe and control group, were established respectively. The test set data of each group was used to test with the corresponding classifier and optimization parameters (Fig. 5).

### Statistical analysis

The clinical data were analyzed by IBM SPSS26 (IBM Corp.). Chi square test was used for counting data. Independent sample t-test was used to verify whether the measurement data conform to the normal distribution, otherwise, Mann Whitney U test was used. P < 0.05 had statistical significance. The efficiency of Auto-ML classifier uses Obfuscation matrix to calculate TPR, TNR, PPV and NPV, draw receiver operating characteristic curve ROC at the same time to get AUC.

## Results

### Patients

Among 219 patients included in the study, 83 were in the severe group and Others were in the moderate group with average age of 52.72 ± 15.45 years and 49.02 ± 16.75 years respectively, the average age of the control group was 50.47 ± 17.25, The proportion of dyspnea and muscle ache in severe group was higher than that in group Moderate (P = 0.000, P = 0.026). However, there was no statistical significance in the analysis of clinical symptoms and laboratory examination data (WBC, LY, ALT, AST, CRP, FIB, Cre and Ure) in the two groups of patients with COVID-19 pneumonia included in the study. The results were shown in Table 1.

### Radiomics's auto-ML model performance and its classifier verification

Figure 5 summarizes the manifestations of the radiomics Auto-ML model in the first CT images of the Non-focus area of COVID-19 pneumonia. The samples of the moderate group, severe group and control group in this study were randomly divided into training set and test set at a ratio of 8:2. In the training set and test set, three classification models were formed, which were moderate group & severe group, moderate group & control group and severe group and control group. In the training set, there are 175 cases in the moderate group & severe group, 189 cases in the moderate group & control group, and 145 cases in the severe group & control group. In the test set, there are 44 cases in the moderate group & severe group, 47 cases in the moderate group & control group, and 38 cases in the severe group & control group. All three groups of data matrix are screened by TPOT pipeline process, Moderate group and Severe group select RandomForestClassifier for analysis, Moderate and Control group select ExtraTreesClassifier for analysis, Severe and Control group select ExtraTreesClassifier for analysis, and provide the best parameters of each classifier for analysis. Note that in Moderate & Control group and Severe & Control group, although the classifier is the same, the optimization parameters are different (Fig. 5).

**Figure 6:**
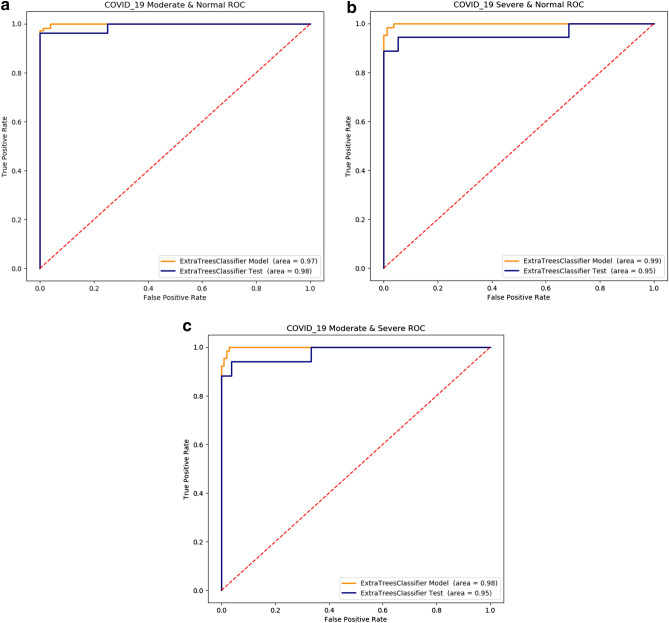
(**a**–**c**) ROC diagrams. The AUC of “moderate” & “severe” training set and test set were 0.98/0.95, respectively (**a**); The AUC of the training set and the test set in the “moderate” & “control” group were 0.97/0.98, respectively (**b**); The AUC of the training set and the test set in the “severe” & “control” group were 0.99/0.95, respectively (**c**).

The training set and test set Obfuscation matrix calculate result of Moderate and Severe group, Moderate and Control group, Severe and Control group were shown in Table 3. ROC curves are shown in (Fig. 6).

**Table 3:** The performance of radiomics auto-ML model in COVID-19 pneumonia 1st CT images.

	TPR (%)	TNR (%)	PPV (%)	NPV (%)	AUC	P-value
**Moderate and severe**						
Training(n = 175)	98.1(107/109)	98.4(65/66)	99.0(107/108)	97.0(65/67)	0.98	0.0001
Test(n = 44)	96.2(26/27)	94.1(16/17)	96.2(26/27)	94.1(16/17)	0.95	0.000
**Moderate and control**						
Training(n = 189)	96.2(76/79)	98.1(107/109)	97.4(76/78)	97.2(107/110)	0.97	0.000
Test(n = 47)	100(20/20)	96.2(26/27)	95.2(20/21)	100(26/26)	0.98	0.000
**Severe and control**						
Training(n = 145)	98.7(79/80)	98.4(64/65)	98.7(79/80)	98.4(64/65)	0.99	0.000
Test(n = 38)	94.7(18/19)	94.4(17/18)	94.7(18/19)	94.4(17/18)	0.95	0.0001

## Discussion

At present, the CT studies of COVID-19 pneumonia are all focused on the focus of pneumonia, there is no study on the non-focus area. As we all know, viral pneumonia is a widespread interstitial inflammation in the lung^20^. In the early stage of pulmonary interstitial inflammation, CT images can hardly to reflect the pathological changes of the lung. Therefore, this study uses the Auto-ML method of radiomics based on CT to study the Non-focus area of COVID-19 pneumonia, in order to find the changes of Non-focus area that CT images cannot find. The study of non-focus tissue in the lung will help clinicians to broader perspective on recognizing COVID-19 pneumonia. Meanwhile it also beneficial to optimize the treatment plan, block the progression of disease, reduce symptoms and improve the cure rate of severe patients. According to the existing data, this is the first time to use CT image-based radiomics to study the non-focus area of COVID-19 pneumonia^2,4,5,9^.

Studies have shown, that the early pathological manifestations of lung injury caused by COVID-19 virus included edema of alveolar epithelial cells and alveolar septum in different degrees, uneven surface of alveoli, and more cytoplasmic vesicles in type I alveolar epithelial cells^21^. These vesicles gradually burst and release fluid, causing morphological changes of alveolar cells, such as cell swelling, deformation, DNA breakage, etc. With the necrosis of the alveolar cells, the pulmonary capillaries further ruptured, resulting in alveolar hemorrhage, pulmonary infection and pulmonary fibrosis. This may be the root cause of severe pneumonia in COVID-19^22^. Radiomics medicine can extract a lot of texture feature information from the image to reflect the heterogeneity of damage. For example, GLCM mainly reflects the characteristics of the internal structure of the image through the change of density^14,20,23^. Therefore, even if no lesions are found on the CT images, we can also analyze different types of texture features extracted to determine whether the lung tissue is damaged. In this study, through the analysis of AUTO-ML classification model, there are significant differences in the texture characteristics of non-focus area in the first CT image between the moderate and severe groups, and there are also significant differences between the moderate and severe groups and the control group, which is similar to the results of Yanling’s study of different types of pneumonia with radiomics^15^.

Different from other radiomics studies, the classification technology of Auto-ML used in this study avoids the limitations of manual selection of machine learning classifiers. Feature selection, feature preprocessing, feature construction, model selection and super parameter optimization^17,18^ are the advantages of TOPT module. Its main code modules are Sklearn and XGBboost, which are commonly used by Auto-ML researchers. According to the results of auto-ML classification of Radiomics, the classifiers used in establishing the classification model of moderate group & severe group are different from those of moderate group& control group and severe group/control group. Although the classification models of moderate group& control group and severe group& control group used the same classifier, the classifiers aim at different models in the calculation process the parameters were optimized. This indicated that TPOT has customized the best model for each data matrix.

In this study, we collected demographic factors, clinical symptoms on admission, and laboratory tests that may be relevant to identification. However, there was no difference between the moderate and severe focus in the early stage of the disease. When the experimental data showed differences, the patient's condition had been aggravated. Therefore, it is an effective way to reduce the rate of severe conversion by effectively predicting the Non-focus area before the patient's condition turns to severe.

In this study, a simple, stable and efficient semi-automatic region growing method, human–computer interaction segmentation method, is selected. Combined with manual modification, the accuracy and repeatability of VOI description are improved. This is of great significance to the accurate segmentation of Non-focus area for feature extraction and model construction. In addition, we chose Non-focus area as VOI. Avoiding the damage of COVID-19 pneumonia, including GGO, consolidation, thickening of broncho-vascular bundle, cystic change and pulmonary vessels and trachea in Non-focus area, which not only avoids the influence of subjective factors, but also can fully measure the severity and degree of lung injury.

However, limitations still existed. Firstly, 219 cases included in the study, thus samples number was relatively insufficient, while there was a risk of over fitting in machine learning and deep learning. Secondly, the data of this study came from the same institution. Although it is a good radiology model for this institution, it is necessary for more research institutions to carry out data sharing, verification, cooperation thus to establish a more general COVID-19 pulmonary inflammation model. Thirdly, there is no completed biological explanation of radiomics features in this study which showing further exploration is needed in the future.

In conclusion, the authors believe that the Radiomics Auto-ML classification model based on the analysis of Non-focus area in the first chest CT image of COVID-19 pneumonia can effectively classify the clinical types of COVID-19 pneumonia.

## Supplementary information

Supplementary Information 1.

Supplementary Information 2.
