# Supplementary material for: The study of automatic machine learning base on radiomics of non-focus area in the first chest CT of different clinical types of COVID-19 pneumonia
Source: Sci Rep. 2020 Nov 3;10:18926. doi: 10.1038/s41598-020-76141-y (PMC7641115; doi:10.1038/s41598-020-76141-y)
Supplement: Supplementary file 1 — Supplementary Information 1. [file 41598_2020_76141_MOESM1_ESM.docx]

**The study of automatic Machine learning base on radiomics of non-focus area in the first chest CT of different clinical types of COVID-19 pneumonia**

Hui-BinTan^1^，ORCID: 0000-0002-4426-3991

Fei Xiong^1^, ORCID:0000-0001-7839-4271

Yuan-Liang Jiang^1^, ORCID: 0000-0002-5278-8384

Wen-Cai Huang^1^, ORCID: 0000-0001-7977-8349

Ye Wang^1^, ORCID:0000-0002-0120-8853

Han-Han Li^1^, ORCID:0000-0002-6844-7314

Tao You^1^, ORCID: 0000-0001-6812-5748

Ting-Ting Fu^1^, ORCID:0000-0001-8173-8389

Ran Lu^1^, ORCID:0000-0003-0490-6240

Bi-Wen Peng^2^, ORCID:0000-0002-5599-6779

1.Department of Radiology, PLA Central theater General Hospital of Chinese

2. School of Basic Medical Sciences, Wuhan University

Address correspondence to:

Corresponding author:

Name: Fei Xiong

Department of Radiology, PLA Central theater General Hospital, Wuhan, Hubei, China

430060

E-mail: [4838524@qq.com](mailto:4838524@qq.com)
